# Cytological diagnosis of peritoneal endometriosis

**DOI:** 10.4103/0970-9371.70757

**Published:** 2010-04

**Authors:** Sudheer Arava, Venkateswaran K Iyer, Sandeep R Mathur

**Affiliations:** Department of Pathology, Cytopathology Laboratory, All India Institute of Medical Sciences, New Delhi, India

Sir,

Endometriosis is a common disease characterized by ectopic growth of endometrial tissue that responds to hormonal stimulation. It primarily affects women in the reproductive age group with mixed symptomatology. Cytological features of endometrial cells in peritoneal fluid are not well described, which we wish to highlight.

**Figure 1:**
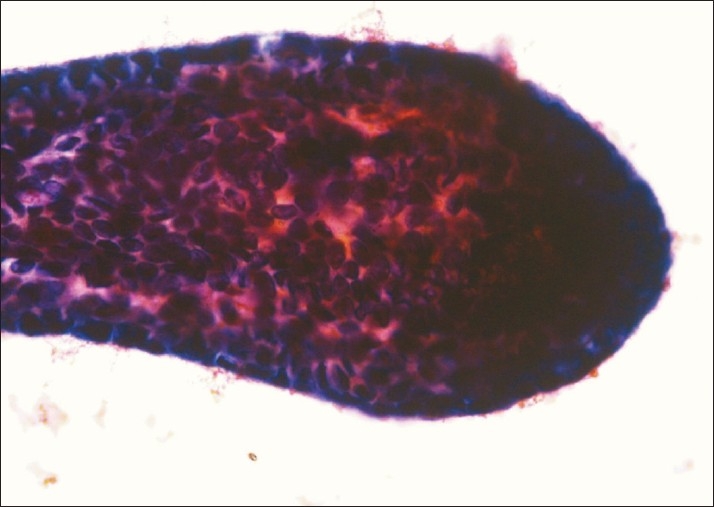
Large endometrial fragment in peritoneal wash fluid showing epithelial lining on the outside, seen on the sides and as honeycombed cells as well as compact stromal cells within (PAP, ×400)

**Figure 2:**
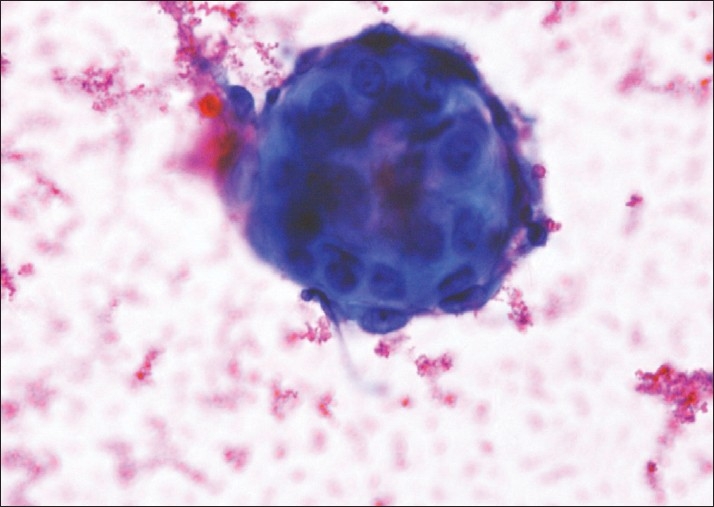
Small spherule of endometrial cells in three-dimensional arrangement, which has to be differentiated from reactive mesothelial cells (PAP, ×400)

A 30-year-old woman presented with lower abdominal pain and dysmenorrhoea of 1 year duration. On examination, she had mild ascites. Bilateral cysts were seen near the fallopian tube on laparoscopy. Diagnostic considerations included neoplastic process or pelvic endometriosis. A peritoneal wash was performed and 100 mL of haemorrhagic fluid was sent for cytological examination. On microscopy, the fluid showed the presence of endometrial cells. The glandular epithelial cells were arranged in variably sized spheres in which the periphery showed epithelial cells, along the border and as a honeycombed sheet, with a cluster of stromal cells in the centre having hyperchromatic nuclei, scant cytoplasm and indistinct cytoplasmic borders [Figure 1]. There was no nuclear atypia and the nucleoli were inconspicuous. Some of the fragments were arranged in a small ball-like configuration in which there was cytoplasm resembling mesothelial cells [Figure 2]. Their three-dimensional nature and tight grouping set them apart from the loose sheets of mesothelial cells of the peritoneal wash. A few hemosiderin-laden macrophages were identified. A cytological diagnosis of endometriosis was made.

The utility of peritoneal wash cytology for diagnosis of endometriosis has been reported.[1,2] In most cases, only hemosiderin-laden macrophages are identified.[2–5] The presence of endometrial cells is more specific but less sensitive than hemosiderin-laden macrophages for the diagnosis of endometriosis. The endometrial cells have been reported in 25%–52% of peritoneal washes done in endometriosis.[1,5] However, recognition of endometrial cells as well as hemosiderin-laden macrophages is essential for diagnosis on morphological basis alone.[6,7] The descriptions, diagnostic features and illustration of endometrial cell morphology in the peritoneal wash fluid is restricted to a few reports.[3,6] The distinction of endometrial cells from mesothelial cells and adenocarcinoma cells is important. Endometrial cells come in three-dimensional fragments that do not resemble mesothelial cells architecturally in washings which are arranged in sheets. However, endometriosis may elicit reactive mesothelial proliferation, which form three-dimensional fragments with calcific concretions, which are difficult to distinguish from endometrial cells.[5] Larger endometrial cell fragments may include stromal cells, which is a useful feature for differentiation as seen in the present case. Presence of hemosiderin-laden macrophages is indirect evidence of endometriosis. In the presence of an adnexal mass, a well-differentiated adenocarcinoma is always a diagnostic consideration and needs to be ruled out by the absence of atypia in the small cells and nuclei of endometrial cells. This distinction becomes difficult if there is reactive mesothelial proliferation in case of endometriosis.[2,5] The use of cell blocks and immunocytochemistry for epithelial and mesothelial cell markers can greatly aid diagnosis in distinction between reactive mesothelial fragments on one side and endometriosis and adenocarcinoma on the other.[2] Correlation with clinical and operative features is essential before cytological diagnosis.
